# Multimodal imaging quality control of epithelia regenerated with cultured human donor corneal limbal epithelial stem cells

**DOI:** 10.1038/s41598-017-05486-8

**Published:** 2017-07-11

**Authors:** Marco Lombardo, Sebastiano Serrao, Vanessa Barbaro, Enzo Di Iorio, Giuseppe Lombardo

**Affiliations:** 1 Fondazione G.B. Bietti IRCCS, Via Livenza 3, 00198 Roma, Italy; 2 Fondazione Banca degli Occhi del Veneto, Via Paccagnella 11, 30174 Zelarino (Ve), Italy; 3 Università degli Studi di Padova, Dipartimento di Medicina Molecolare, Via A. Gabelli 63, 35121 Padova, Italy; 4 Consiglio Nazionale delle Ricerche, Istituto per i Processi Chimico-Fisici, CNR-IPCF, Viale F. Stagno D’Alcontres 37, 98158 Messina, Italy; 5 Vision Engineering Italy srl, Via Livenza 3, 00198 Roma, Italy

## Abstract

Current imaging techniques for the characterization of differentiated corneal limbal stem cells are destructive and cannot be used in eye bank for monitoring the regenerated epithelium in culture. We presented a minimally invasive, multimodal, marker-free imaging method for the investigation of epithelia regenerated with cultured human donor corneal limbal epithelial stem cells. Two-photon fluorescence and harmonic generation signals were collected from specimens in culture and used for evaluating the structure and morphology of epithelia cultured on two different bio-scaffolds; in addition, donor human corneal tissues were used as controls. The method provided reliable information on the organization of cellular and extracellular components of biomaterial substrates and was highly sensitive to determine differences between the density packing arrangement of epithelial cells of different biomaterials without relying on inferences from exogenous labels. The present minimally invasive standardized quality control methodology can be reliably translated to eye banks and used for monitoring harvested corneal limbal stem cells growth and differentiation in bioengineered materials.

## Introduction

In stem cell research, there is a high demand on techniques for the minimally-invasive, marker-free observation of growth, proliferation, differentiation, and stability of living stem cells under near-physiological conditions^1–6^. The development and validation of reliable means for monitoring regenerated corneal epithelial cells using entirely endogenous sources of contrast would represent a significant challenge in efficiently controlling stem cell growth and differentiation in tissue engineering and stem cell therapy for eye care.

Limbal stem cells deficiency (LSCD) has been defined as the destruction or dysfunction of the stem cells containing limbal epithelium, leading to failure of corneal epithelial regeneration, accompanied by chronic inflammation, stromal scarring, neovascularization and persistent epithelial defects. The disease leads to visual loss due to severe corneal opacity and is associated with high risk of corneal graft failure from rejection exceeding 35% at three years^7^.

Current imaging techniques for the characterization of differentiated corneal limbal stem cells include immunocytochemistry, immunofluorescence microscopy and electron microscopy. The analyzed specimens, however, cannot be used for further operations due to the destructive effects of the examination procedures that require exogenous markers and cannot be used in eye bank for monitoring the regenerated epithelia during culture. The greatest advantages of two-photon optical microscopy over immunofluorescence and electron microscopy imaging include no requirements for fixation and dyes, molecular specificity, decreased out of focus photodamage, increased imaging depths and intrinsic optical sectioning, which ultimately provides the possibility of three-dimensional (3D) reconstruction of the anisotropic structural organization of tissues. The reliability of two-photon optical microscopy to image differentiated stem cells has been formerly shown by Uchugonova and König^8^.

In this study, we present a minimally-invasive approach based on two-photon optical microscopy for investigating the epithelia regenerated from human corneal epithelial limbal stem cells on different bio-scaffold substrates, such as the *hemicornea* and fibrin gel^7, 9^. The *hemicornea* is a human-derived bioscaffold and consists of a donor anterior corneal stromal lenticule; the fibrin gel is the golden standard for cultivating human limbal epithelial stem cells and treating patients with LSCD^7, 9, 10^. Data of regenerated epithelia and their substrates were compared with human donor eye bank corneoscleral tissues with intact epithelium and de-epithelialized anterior corneal stromal lenticules, which were used as controls.

## Results

**Figure 1:**
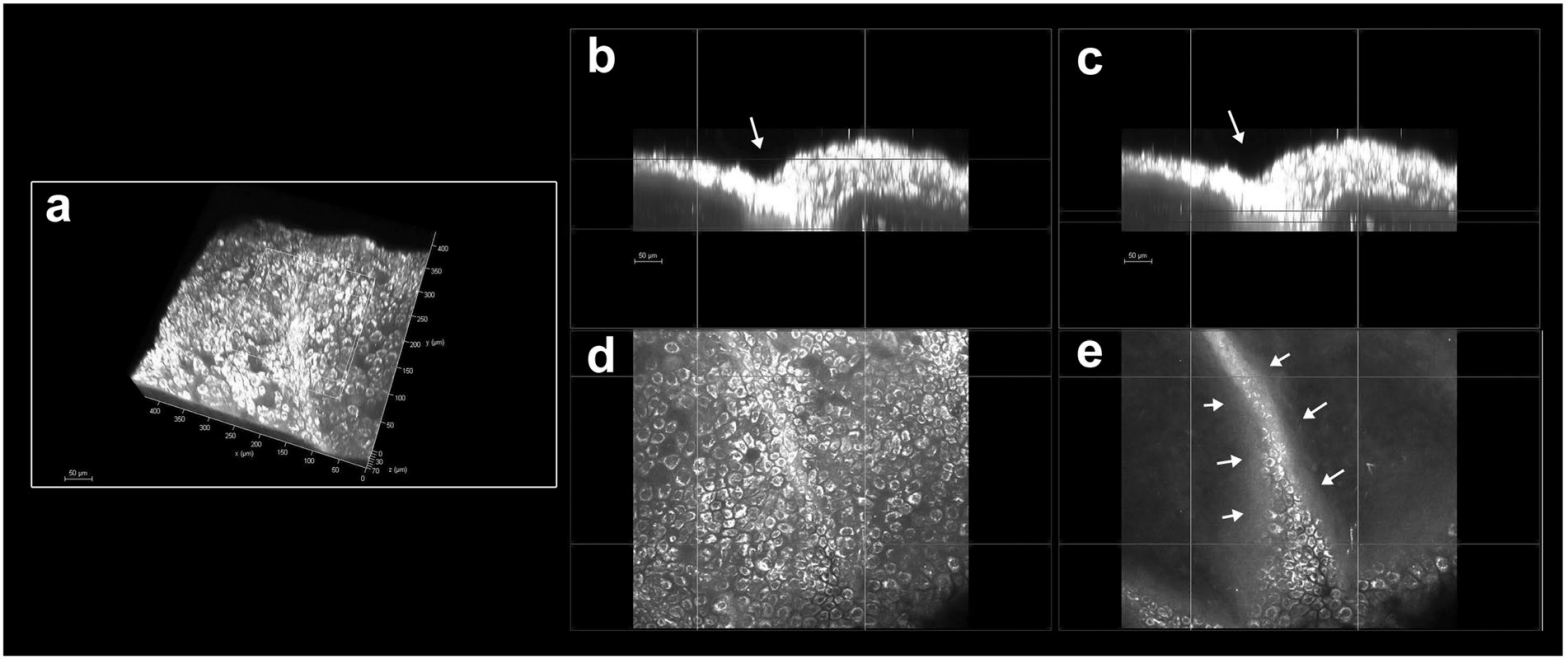
Pseudo-palisade of Vogt in the *hemicornea*. (**a**) Three-dimensional (3D) reconstruction of the *hemicornea* (TPEF signal) showing the pseudo-palisade of Vogt (scale bar 50 µm). Cross-sections (**b** and **c**; 400 × 60 µm) and *enface* (**d** and **e**; 400 × 400 µm) images of the *hemicornea* at different depths highlighting the intrastromal digital invasion by basal epithelial cells (arrows). This structure was randomly observed in scattered regions the *hemicornea*. The palisades of Vogt are distinctive features of the adult corneoscleral *limbus* and represent the specialized microenvironment (niche) of corneal stem cells.

**Figure 2:**
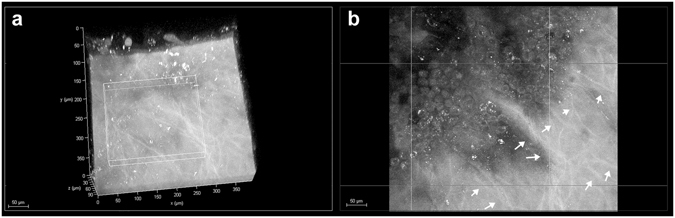
Basal nerve plexus in the *hemicornea*. (**a**) 3D reconstruction of the *hemicornea* (TPEF signal) showing the basal nerve plexus (scale bar 50 µm). (**b**) The nerve fiber bundles (arrows) run parallel to each other forming thin branches that penetrate into the basal epithelial plane. The corneal epithelium is one of the most highly innervated structures in the human body; proper innervation is necessary for maintenance of normal corneal functions. There is strong evidence from laboratory studies that the nerve fibers provide trophic support to the corneal epithelium.

**Figure 3:**
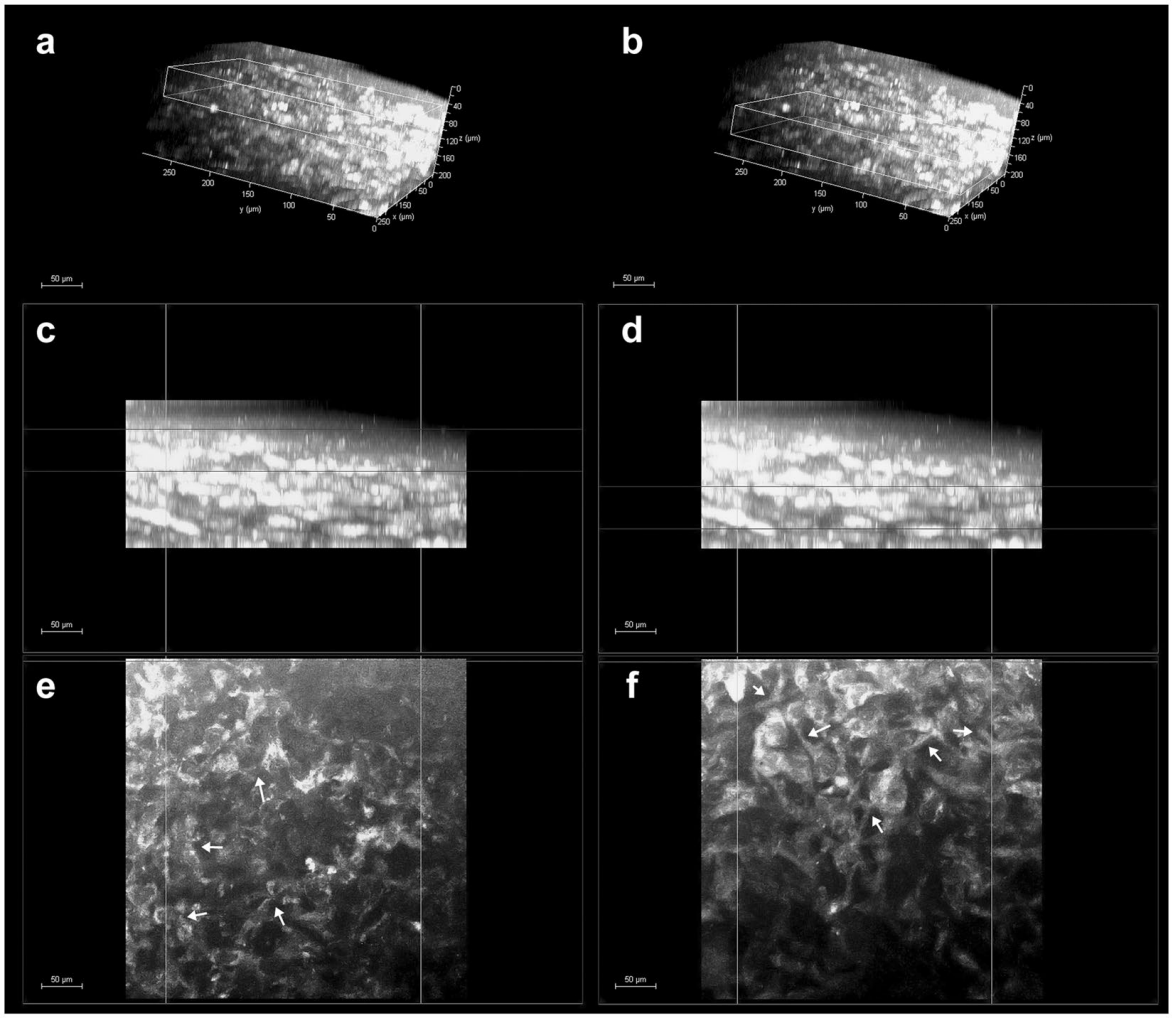
Corneal stroma keratocytes in the *hemicornea*. (**a** and **b**) 3D reconstruction of the *hemicornea* (TPEF signal) showing the stromal keratocytes at different depths (scale bar 50 µm). The white box in panel A encloses the anterior stroma ranging between 40 and 90 µm depth (**c** and **e**); the white box in panel b encloses the stroma ranging between 120 and 170 µm depth (**d** and **f**). The panels c, d and e, f show the cross-section and *en face* images of the *hemicornea* stroma respectively. The stromal keratocytes show a dendritic-like morphology with processes connecting the cells to each other forming cellular networks (syncytium; arrows).

**Figure 4:**
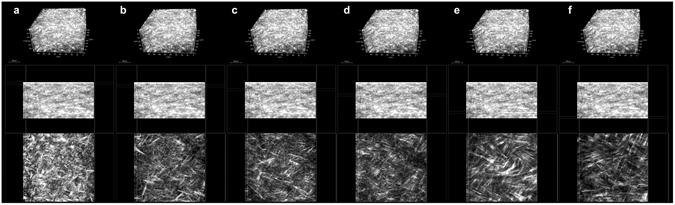
Organization of anterior stromal collagen fibers in the human cornea. (Upper row) 3D reconstruction of the anterior 250 µm stroma of the human cornea (forward SHG signal) showing the depth-dependent organization and arrangement of collagen fibers (scale bar 100 µm). Middle row) cross-section images of the corneal stroma; from the left to right, the boxes enclose regions of the stroma at varying depths, ranging from the most anterior stroma underlying the Bowman’s layer to 250 µm depth. (Lower row) corresponding *enface* images of the stroma. In the most anterior stroma (**a**), the collagen fibers are arranging in tiny and short bundles densely intertwined at different planes; they arrange in thin and densely packed lamellae intersecting each other across 100- and 150-µm depth (**b**, **c** and **d**); these lamellae become increasingly wider and thicker with increasing depth. From ≥200 µm depth, the collagen lamellae shows a grid-like structure, crossing each other at almost vertical angles (**e** and **f**).

All specimens were imaged without the need of labelling. In the *hemicornea*, the regenerated epithelium resembled the normal architecture of the human cornea; the resulting epithelium was stratified into three to five cell layers, with basal cuboidal cells differentiating upward to wing cells. In all cases, the basal epithelial plane was firmly attached to the underlying Bowman’s layer; in 50% of specimens, it showed some digital invasions of the limbal basal epithelium in the palisades of Vogt (Fig. 1 and Supplementary Video 1)^11^. The sub-basal nerve plexus could be resolved in the *hemicornea* and consisted of a network of tiny fibers entering the basal epithelial layer (Fig. 2). The stromal keratocytes of *hemicornea* showed a dendritic-like morphology and formed a syncytium throughout the stromal depth (Fig. 3)^12^. High-resolution images of the *hemicornea*’s stroma were collected both in forward (F-SHG) and backward (B-SHG) directions. The collagen fibers of the *hemicornea* were regularly arranged and intertwined with each other, as found in the normal human cornea (Fig. 4).

**Figure 5:**
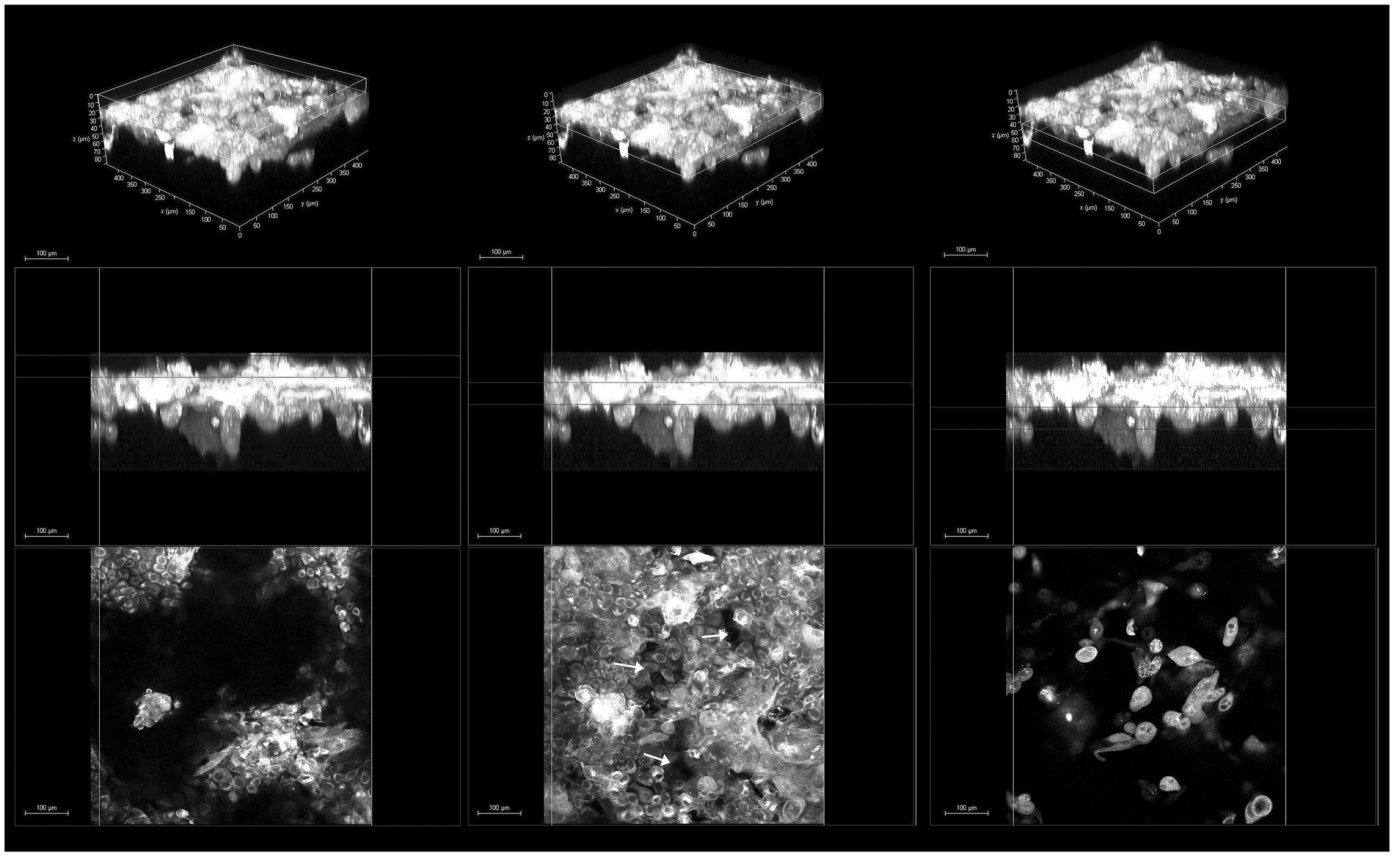
Regenerated epithelium on fibrin gel. (Upper row) 3D reconstruction of the fibrin gel (TPEF signal) with cultivated limbal corneal epithelial cells (scale bar 100 µm). The white boxes highlight the characteristics of regenerated epithelium, which are shown in the middle (cross-section images) and lower (*enface* images) rows at different depths respectively. Middle rows) abnormal stratification of the regenerated epithelium was found in all specimens. (Lower rows) the cells show also variations in shape and size across depth and are not confluent even at the basal plane (arrows). Neither TPEF nor SHG signals could be collected from fibrin scaffolds.

The epithelial layer stratification and cell morphology in fibrin gel were significantly different from *hemicornea*. Inhomogeneous stratification and hyperproliferation of epithelial cells were observed in fibrin scaffolds; by visual inspection, the cells showed differences of shape and size across depth and were not firmly attached to each other, even at the basal layer (Fig. 5). No pseudo-palisades of Vogt were seen in any fibrin gel; no SHG emission spectra were recorded from fibrin scaffolds.

The cell density was 4236 ± 760 cells/mm^2^, 2925 ± 643 cells/mm^2^ and 6806 ± 436 cells/mm^2^ for the basal epithelial layer of the *hemicornea*, fibrin gel and control corneal tissue respectively; the cell spacing was measured to be 12.8 ± 1.1 µm, 15.1 ± 1.8 µm and 9.6 ± 0.5 µm for the basal epithelial cells respectively. The density and spacing of cells were significantly different between the bio-scaffolds and the control corneal tissues (P < 0.001); differences between the two bio-scaffolds were also statistically significant (P < 0.01). The percentage of hexagonal Voronoi tiles at the basal epithelial layer was 41 ± 7%, 37 ± 10% and 48 ± 6% respectively. The differences between the fibrin gel and control corneal tissue approached statistical significance (P = 0.05).

The mean TPEF signal intensity of the basal epithelial layer was 118 ± 29 au, 52 ± 12 au and 112 ± 47 au for the *hemicornea*, fibrin gel and corneoscleral tissue respectively. The difference in the results was statistically significant between the *hemicornea* and fibrin gel (P < 0.001) and between the fibrin gel and control corneal tissue (P < 0.01). The mean TPEF signal intensity of the keratocytes lying in the most anterior stroma beneath the Bowman’s layer was 63 ± 10 au and 50 ± 12 au in the *hemicornea* and control corneal tissue (P = 0.14) respectively.

**Figure 6:**
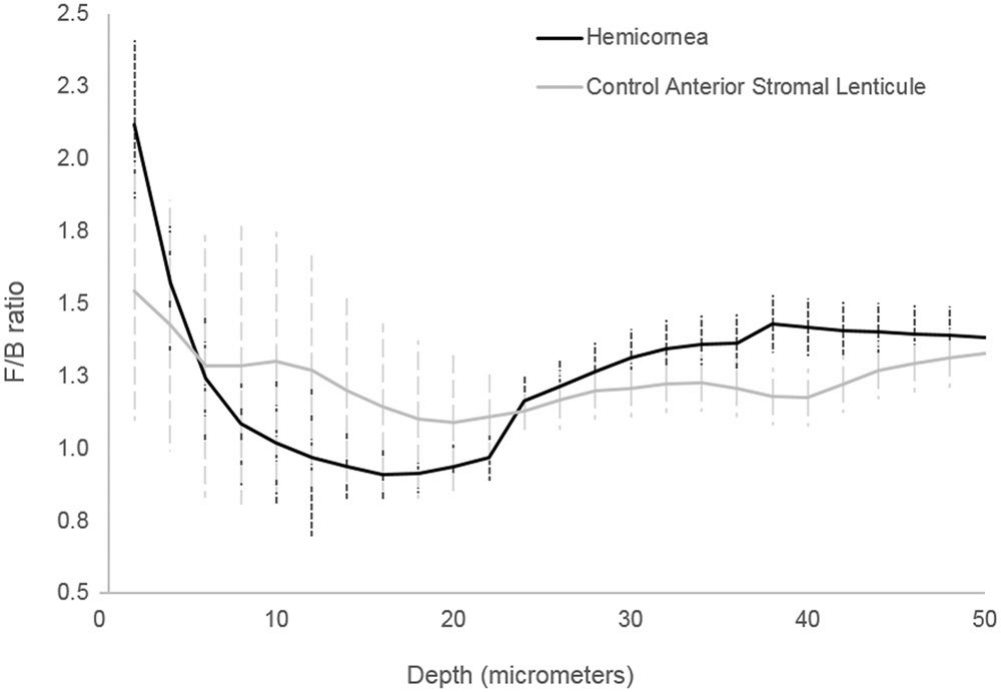
F/B ratio of the *hemicornea* and control anterior stromal lenticule. Averaged measured forward/backward SHG intensities as a function of depth (from the Bowman’s layer to 50 µm depth) for the *hemicornea* (black curve) and control anterior stromal lenticules (grey curve). The vertical lines represent ± SD.

The high dispersion (i.e., low coherency values) of the collagen fiber orientation distribution was attributed to the highly intertwined arrangement of these fibers crossing each other at variable angles in the most anterior corneal stroma. The arrangement of collagen fibers in the *hemicornea* showed relatively higher coherency values than the anterior stromal lenticules that were used as controls; the average coherency values were 0.24 ± 0.04 and 0.21 ± 0.07 (P = 0.03) respectively. Backward SHG images provided similar information as forward SHG images in terms of orientation for the stromal collagen fibers with differences within 15 degrees up to 65 µm stromal depth in the *hemicornea* (mean Δγ = 4.4 ± 5.3 degrees) and control anterior stromal lenticule (mean Δγ = 4.0 ± 7.0 degrees) respectively. The forward to backward second harmonic generations ratio, F/B, showed comparable values between the *hemicornea* and control anterior stromal lenticules, except for the most anterior 20 µm depth (Fig. 6). The main results of this work were summarized in Table 1.

**Table 1:** Main results (mean ± SD) of the two-photon fluorescence (TPEF) and second harmonic generation (SHG) signals used for evaluating the *hemicornea* and fibrin gel in comparison with donor human corneal tissues.

	Corneal tissues (control; n = 3)	Anterior stromal lenticules (control; n = 3)	Hemicornea (n = 6)	Fibrin gel (n = 3)
Epithelial cell density (cells/mm^2^)	6806 ± 436*^,#^	N.A.	4236 ± 760^†^	2925 ± 643
Epithelial cell spacing (µm)	9.6 ± 0.5*^,#^	N.A.	12.8 ± 1.1^†^	15.1 ± 1.8
Epithelial cell packing arrangement (% of 6n)	48 ± 6^#^	N.A.	41 ± 7	37 ± 10
TPEF epithelium (a.u.)	112 ± 47^#^	N.A.	118 ± 29^†^	52 ± 12
TPEF stroma (a.u.)	50 ± 12*	N.A.	63 ± 10	N.A.
Coherency	N.A.	0.21 ± 0.07*	0.24 ± 0.04	N.A.
Δγ (degrees)	N.A.	4.0 ± 7.0	4.4 ± 5.3	N.A.

6n: hexagonal Voronoi tiles.

N.A.: not applicable.

*P ≤ 0.05 between control and *hemicornea*.

^#^P ≤ 0.05 between control and fibrin gel.

^†^P ≤ 0.05 between *hemicornea* and fibrin gel.

## Discussion

To the best of our knowledge, this is the first study reporting the feasibility and reliability of two-photon optical microscopy to investigate the epithelia regenerated by human corneal epithelial limbal stem cells on different biomaterials. The complementary collection of SHG and TPEF emission spectra was valuable to obtain marker-free, quantitative information on fresh regenerated epithelia of cultured human corneal limbal epithelial stem cells. The epithelial architecture of the *hemicornea* was almost comparable to the native human corneal tissue, though the preferred cell packing density arrangement of the basal layer showed values lower than controls. Data of control donor tissues were in fair accordance with previous *in vivo* study evaluating the corneal basal epithelial layer of adult subjects^13, 14^; using confocal corneal microscopy, the size and density of the basal epithelial cells in the peripheral cornea of living healthy subjects have been found to be 10.1 ± 0.8 µm and 7120 ± 362 cells/mm^2^ respectively^13, 14^. As far as we know, based on a thorough literature search, no data on cell density of the epithelia grown onto fibrin gels have been previously reported. Overall, the present method showed significant differences in the packing density arrangement of epithelia grown on different biomaterial substrates; it may be valuable to assess novel techniques of stem cell therapy, as they will become available.

High-resolution TPEF images of stromal keratocytes were collected from all *hemicorneas*; we were also able to resolve the anterior stromal nerve fibers, which have not been observed before in biomaterial substrates, entering the basal epithelial layer of the regenerated epithelium of *hemicornea* ^15^. Based on current knowledge, the stroma of *hemicornea* could represent an active matrix for supporting, through cell-to-cell communication, all the aspects of the overlying epithelial cells’ growth and differentiation^15^. Although the role of the mesenchyme in the initial establishment of the histo-architecture and its instructive influence on the developing overlying epithelial structures has been well established only during embryonic development^15, 16^, tissue-specific differentiation of the epithelial corneal phenotype could be probably determined by a combination of stroma-mediated mechanisms and intrinsic epithelial properties even in adult and post-mortem tissues.

Analysis of second harmonic generation signals was done in order to characterize the main structural corneal stroma components, i.e., the collagen fiber, and how their organization and arrangement vary through the thickness^17, 18^. The results were in accordance with previous studies showing the high dispersion of orientation distribution of collagen fibers through the anterior human corneal stroma^19, 20^. The complementary information provided by the two parameters, i.e. the preferred orientation of collagen fibers and the coherency value, which gives a measure of the degree of order of fibers aligned along this preferential direction, may be valuable for understanding the structural integrity of the most anterior stroma^21, 22^, which supports epithelial cells’ sliding movements and stratification. The present method provided information on the preferred orientation of stromal collagen fibers from the backward SHG images that were consistent with forward SHG images. This was verified both in the *hemicornea* and control anterior stromal lenticules. Based on these results, analysis of backward SHG images only may be valuable for monitoring the structure of the *hemicornea* stroma, because this will be the most likely configuration for the possible future diagnostic implementations of the technique.

Since collagen fibrils are the primary cause of scattering in corneal stroma, the structural differences in collagen organization and arrangement probably contributed to the F/B ratio differences in the most 20 µm anterior stroma between samples. Specifically, differences in the extrafibrillar spacing and collagen fibrils as well as in collagen fibers’ arrangement (as depicted by the statistically significant different coherency values between samples) could contribute to variable scattering between samples^23–25^. It could not be excluded that tissue manipulation during epithelial stem cell cultivation may have an influence in the structural differences of the most anterior stroma^25^.

The corneal stroma lenticules of *hemicornea* had 200 µm targeted depth and allowed us to collect more efficiently the SHG signal emitted in the backward direction. For collagenous specimens with thickness of *λ*/4 (*λ* is the excitation wavelength), whose molecular distribution possesses an axial periodicity in the vicinity of *λ*/4, up to 25% of the SHG power is radiated in backward directed^23, 26^. In addition, the radiated SHG power in the backward direction increases when the size of the scattering structure along the optical axis is much lower than λ and also because collagen fibrils in the corneal stroma are less aligned with respect to the optical axis^26, 27^. SHG microscopy can be also configured to extract more information on the collagen architecture and preferred organization throughout stromal depth through polarisation resolved methods^21^. It should be object of further investigation if this methodology could be used to detect the biosynthesis (fibrillogenesis) or remodelling of collagen in bio-engineered scaffolds^1, 23, 28, 29^.

Since the main scope of this work was to thoroughly identify and characterize the structure and morphology of cellular and extracellular components of the bio-scaffolds, we could not collect information on the metabolic information of the regenerated epithelia. On the other hand, the present methodology could be enhanced, through the careful selection of combinations of excitation wavelengths (e.g., 755 nm and 860 nm) and selective emission filters (e.g., 460 nm and 525 nm), to identify the intrinsic cellular fluorophores (e.g., NADH and FAD) and achieve metabolic information^5, 6, 13, 30, 31^. In addition, two-photon fluorescence lifetime imaging has been already shown to be valuable for real time monitoring of stem cell differentiation^8^. Collectively, both intensity-based and lifetime-based image analysis would make two-photon microscopy a powerful tool for quantitatively monitoring of epithelial stem cell differentiation during *in vitro* cultivation in order to optimize culture conditions, though the potential risk of photodamage for repeated scans of the sample over time should be assessed^32^. In this study, no change of epithelial cell morphology or other adverse effects were observed after repeated scanning.

Current management of unilateral LSCD is carried out using a complex multistage approach that requires several years for its fulfilment and definite restoration of vision, though in less than 60% of cases. This approach includes using autologous limbal stem cell transplantation grown onto fibrin gel to regenerate the corneal epithelium followed by penetrating keratoplasty to repair scarring to the anterior corneal stroma, which is often caused by caustic burns. The *hemicornea* would repair both the epithelial and stromal damage (anterior stromal scarring is most commonly associated with superficial neovascularization) with a “one step” surgical procedure thus saving time and improving patient care.

In conclusion, the present methodology can be helpful to gain information on growth and differentiation of regenerated epithelia under near-physiological conditions. Miniaturization of the technology (e.g., using fiber optics and more compact pulsed IR laser sources) would greatly hasten the translation of two-photon optical microscopy in the eye bank as a valuable tool to establish and standardize quantitative, minimally-invasive, quality control methodology for harvested corneal limbal stem cells growth and differentiation. In addition to investigation for research and clinical purposes, the present methodology may be helpful for developing valid alternative method for assessing safety and toxicity of drugs and medical devices by imaging human corneal bio-engineered tissues, such as the *hemicornea*, as innovative *in vitro* organotypic culture systems^33^.

## Methods

The study was conducted in compliance with the guidelines of the Declaration of Helsinki for research involving the use of human tissues and with the guidelines for the clinical translation of stem cells of the International Society for stem cells. Written informed consent from the next of kin was obtained for the use of samples in research. The laboratory study was approved by the local ethical committee (Provincia di Venezia, Italy). Donors did not have history of corneal pathologies, eye surgery or any major systemic diseases. The scaffolds were prepared in the Fondazione Banca degli Occhi del Veneto (Venezia Zelarino, Italy), as fully described in previous work^7, 9, 10^. A donor corneoscleral tissue was placed in an artificial anterior chamber and the *hemicornea* was obtained by microkeratome resection of an anterior stromal lenticule with 200 µm targeted depth. Thereafter, limbal corneal keratinocytes were plated onto the *hemicornea* and cultured under submerged conditions for 7 days and air lifted for 14 further days; the corneal epithelial cells that were plated on fibrin gel were cultivated at the air-liquid interface for 14 days. The submerged culture technique has been shown to prevent early differentiation and retain more stem cells as opposed to the stratification and differentiation that is observed in extended airlift culture techniques, and to provide highly reproducible outcome (i.e., the eye bank technicians had high success rate, ≥70%, in making and harvesting cell sheets with similar morphologic characteristics in both biomaterial substrates)^7, 9^. Donor corneoscleral tissues with intact epithelium and 200 µm targeted depth anterior stromal lenticules prepared by microkeratome resection were used as controls.

The tissues and biomaterials used in this work included six *hemicorneas* and three fibrin gels with cultivated epithelial limbal stem cells, three donor corneoscleral tissues with intact epithelium and three donor anterior stromal lenticules.

### Two-photon microscopy imaging

The two-photon microscopy set-up used in this study was based on a Leica DM6000CS (Leica Microsystems GmbH, Germany) upright microscope. A tunable Ti:sapphire laser (VISION II, Coherent, CA, USA) with an integrated proprietary prism-based unit designed to compensate the broadest range of Group Velocity Dispersion was used as excitation source to perform two-photon emission fluorescence (TPEF) and second harmonic generation (SHG) axial scanning measurements in all specimens. This laser presents a tunable wavelength range from 680 to 1080 nm, operating with a pulse width of 140 fs at 80 MHz of repetition rate. The laser power was attenuated by an Electro-Optical-Modulator (EOM) and then coupled into the Leica SP8-Spectral Scan-Head (Leica Microsystems GmbH, Germany) where it passed through the x-y scanning module, allowing the scanning in the x-y focal plane, before being focused by a HCX IRAPO 25x/0.95 NA, water immersion objective with a working distance of 2.5 mm (Leica Microsystems GmbH, Germany). Such lens was ideal for deep tissue imaging at high-resolution, with high transmission in visible and infrared ranges, and axial and lateral colour correction for multiphoton excitation. The whole system was enclosed in a black plastic box in order to assure eye-safety for any IR scattering light from the tissue samples.

Each specimen was placed upward on a quartz microscope slide under the microscope and illuminated by the laser tuned to 712 nm or 810 nm for collecting the TPEF or the SHG signals from cellular and extracellular components of each sample respectively. For each sample, the TPEF and SHG signals were collected consecutively in the same imaging session. The laser power was 15 mW before entering the water immersion objective in all sessions; it was measured before and during each session of measurements in order to allow direct comparison of measurements between different sessions of experiments^34^. The TPEF light was collected in backward direction by a nondescan detector (NDD) for reflected light and the SHG signal was collected both in backward and forward directions by a pair of NDDs in the transmission and reflected paths of the microscope respectively. For the transmission NDD unit, the incoming light first went through a short pass filter (λ < 680 nm, SP680) and then was filtered by a 10 nm FWHM band pass filter centered at 405 nm (FF01-405/10-25, Semrock Inc., Rochester, NY, USA) in order to image only the SHG forward signal emitted by the corneal stroma. The reflected light coming from the sample was first filtered by an IR filter SP680 and then encountered a dichroic beam splitter (Di02R405-25x36 Semrock Inc., Rochester, NY, USA). Each of the transmitted and the reflected light paths from the dichroic entered to each of the two reflection NDDs; the reflected light was filtered by a band pass filter 447/60 (Semrock Inc., Rochester, NY, USA) and entered into the reflection NDD unit and the transmitted light was again filtered by FF01-405/10-25 to provide the backward SHG signal.

In order to compare data between different sessions of experiments, the NDDs sensitivity settings for collecting the TPEF and SHG signals were the same for all specimens. The TPEF emission spectra collected with this configuration were consistent with the literature values for NAD(P)H and flavins, enabling visualization of cellular structures (mitochondria)^6, 16, 27^. Under 2-photon excitation at 700–750 nm wavelengths, tissue fluorophores are primarily derived from the aromatic amino acids such as tryptophan, tyrosine and phenylalanine which emit at 400–600 nm wavelengths. The TPEF enabled visualization of epithelial and stromal cells and nerve fibers. The SHG emission spectrum (400–430 nm) is consistent with collagen, which holds noncentrosymmetric structure, excited at wavelength between 800–860 nm^17, 18, 29, 31^.

Each sample was scanned twice for collecting either TPEF or SHG signals with a 2 µm or 4 µm step size in the *z*-axis, extending from above the epithelial surface to below the anterior 200 µm stroma; images with 1024 × 1024 pixels resolution (2 frames average) were recorded on several locations of the *hemicornea* and fibrin gel and in the central and para-central regions of the corneoscleral tissue. Image visualization were carried out using proprietary Leica software and an image processing package (Image J, NIH, www.imagej.nih.gov/ij/ *)* and custom-written macros.

### Image analysis

A sampling area of 120 × 120 µm was used to analyse the TPEF images of the basal epithelial layer of the biomaterials and corneoscleral tissues as well as the TPEF signal of the most anterior stromal keratocytes lying at 60 ± 10 µm from the epithelial surface of both the *hemicornea* and control corneal tissue (i.e., beneath the Bowman’s membrane). Cellular fluorescence was measured as the mean intensity of all pixels within the sampling area; in TPEF images of the stroma, the average fluorescence intensity from an area devoid of cells was first measured and subtracted as background using the proprietary Leica software.^32^ Data were exported in a text array for statistics.

The density, spacing and preferred packing arrangements of cells were analysed on several sampling areas of 120 × 120 µm. Image cell labelling process was performed using an algorithm implemented with the image processing toolbox in Matlab (The Mathworks Inc, Natick MA, USA)^35, 36^; cells were selected by two expert investigators (ML and GL) and the results for each sampling window recorded (Supplementary Fig. 1). The x, y coordinates of the cell centroids were then stored in a text array and used to calculate the cell metrics. The number of cells in each sampling area was divided by the epithelial area to derive an estimate of cell density (cells/mm^2^). Cell spacing was calculated as the distance of the closest cell in the array for each of the cell, which is equivalent to the *nearest neighbour distance* (NND; µm)^37^. Cell packing arrangement was analyzed using Voronoi diagrams. This approach is commonly used in computational geometry to subdivide a plane into regions based on distance to point sites^38^. The Voronoi tessellation was implemented by the voronoi Matlab function from the bidimensional coordinates of labelled cells^35, 36^. Each Voronoi cell was coded by a different colour corresponding to the number of their neighbouring cones: gray = tetragonal (4*n*) arrangement, yellow = pentagonal (5*n*) arrangement, green = hexagonal (6*n*) arrangement; blue = heptagonal (7*n*) arrangement and white = octagonal (8*n*) arrangement. The Voronoi regions containing pixels that extended beyond the bounds of each section were excluded from further analysis, thus creating a buffer zone to minimize the *boundary effect*.

The plugin OrientationJ (http://bigwww.epfl.ch/demo/orientation/) in ImageJ was used for analysis of stromal collagen fiber orientations, based on the evaluation of the structure tensor in a local neighborhood, as previously described by Vielreicher *et al*.^29, 39^. The *Dominant Direction* function was used to calculate the preferred fiber orientation and the coherency values over 120 × 120 µm sampling area for each SHG (both forward and backward) image stack from the Bowman’s layer to a maximum of 65 µm in the anterior stroma. Coherency, *C*, was used as a measure for the local degree of order of fibers (0 = isotropic symmetry/disordered system of fibers; 1 = anisotropic symmetry/ordered system along the dominant direction).

The preferred fiber orientation, computed by calculating the structure tensor and its associated eigenvalues, corresponds to the direction of the eigenvector associated to the smallest eigenvalues of the tensor, i.e λ_min_, i.e., whose orientation is perpendicular to the maximum direction of the gradient^22^. For each SHG image, we extracted the fiber orientation distribution (Supplementary Fig. 2) obtained by evaluating the preferred fiber orientation for each pixel and then computing the histograms of the orientations using 180 equally spaced bins.

Coherency is a measure of confidence or anisotropy index, defined as:

$$C=0\le \frac{{\lambda }_{max}-{\lambda }_{min}}{{\lambda }_{max}+{\lambda }_{min}}\le 1$$

If *C* ≈ 0, which corresponds to *λ*_*max*_ = *λ*_*min*_, then the region of interest is rotational symmetric without any dominant direction, the structure has no preferred orientation and collagen fibers are distributed in each direction. If *C* ≈ 1, which corresponds to *λ*_*max*_ > 0, *λ*_*min*_ ≈ 0, the fibers in the region of interest are well-aligned and a preferred orientation exists. For 0 < *C* < 1, the preferred fiber orientation lies between the gradient directions. In general, a coherency close to 1 indicates that the structure holds a preferred orientation and spatial anisotropy exists in the region of interest; a coherency value close to 0 indicates that there is no preferred fiber direction and isotropy symmetry exists.

The difference of the preferred fiber orientation (Δγ) for the forward and backward SHG images was used as a parameter for comparison between SHG signals^40^.

The measurement of the forward and backward SHG intensities has been shown to be valuable for collecting information on the structure of fibrillar tissues, such as the corneal stroma^24^. Owing to its coherent nature, SHG is usually observed to be emitted in the forward (transmitted) direction. If axial scatterers are separated by a multiple of distance of λ_SHG_/2, i.e., λ/4, an appreciable backward (coherent) directed SHG is observable. In addition, the backward SHG signal may also be produced from multiple scattered forward (incoherent) SHG. If perfect phase match occurs, i.e., ΔK = K_2ϖ_  − 2K_ϖ_ = 0, the SHG emission is 100% forward directed and co-propagates along laser beam direction. If imperfect phase match (as usual) is present, it gives rise to a corresponding distribution of forward and backward emitted components and, as a result, SHG in tissues is described as quasi-coherent. In tissue imaging, the measured directionality of the SHG signal (F/B ratio) will comprise a convolution of the initially emitted directionality (F_SHG_/B_SHG_) and the subsequent scattering of these photons at λ_SHG_^24, 41^. The F_SHG_/B_SHG_ is highly dependent upon the fibril diameter, the packing density and regularity relative to the size-scale of the SHG wavelength. The bulk optical properties are related to density (primarily the scattering coefficient, µ*s*) and organization (primarily the scattering anisotropy, *g*) of the fibrillar assembly^24, 41^. Specifically, structures that are ordered on the size of λ_SHG_ in the axial direction will give rise to predominantly forward SHG, whereas the emission from smaller and/or more random structures with larger Δk values will have relatively less forward directed signal and higher backward signals.

It should be kept in mind that F_SHG_/B_SHG_ is not the quantity measured in an experiment, which we define as F/B ratio. The first metric takes into consideration the quasi-coherent interactions associated with fibril diameter, packing density and randomness in the fibrillary matrix^41^. The latter metric, which was calculated as the ratio of the mean (forward and backward) SHG signal intensity for all the frames in each stack, consists of the emitted directionality and components arising from forward and backward scattering of the initially emitted photons. The F/B ratio was calculated in the region of interest (120 µm side) in order to obtain information on the structural differences between the stroma of the *hemicornea* and control anterior lenticules. The greater the F/B ratio, the greater the fibril packing density (if < λ_SHG_) and/or the larger the fibril diameters (approximately λ_SHG_, independent of the packing arrangement)^24, 25, 41^.

We chose to use sample areas of 120 μm side in the central region of each TPEF and SHG images in order to have an area big enough to evaluate the main features of the epithelia regenerated on the two different bioscaffolds and the stromal collagen fiber orientation respectively as well as to avoid any loss of information that may occur at the edges of the images due to the samples’ shape or locally due intrinsic heterogeneity of biological tissues (e.g., filter effects, such as loss of laser intensity and/or loss of SHG signal)^23–25, 41, 42^.

### Statistics

Data were expressed as mean ± standard deviation. Statistics were performed using the SPSS software (SPSS Inc., version 17.0). The normal data distribution was tested by using the one-sample Kolmogorov-Smirnov test. The analysis of variance and the Tukey pairwise test were used to test significance between TPEF signal intensity and cell metrics among biomaterials. Statistical significance was set at *P* < 0.05 for all the tests performed.

Sample size (allocation ratio 2:1) was calculated to determine a mean difference of 1200 (±600) cells/mm^2^ between the epithelia regenerated on the two different biomaterials at a significance level of 5% and a power of 81%.

## Electronic supplementary material

Supplementary info

Supplementary video 1

supplementary video 2
