# Supplementary material for: Multimodal imaging quality control of epithelia regenerated with cultured human donor corneal limbal epithelial stem cells
Source: Sci Rep. 2017 Jul 11;7:5154. doi: 10.1038/s41598-017-05486-8 (PMC5506064; doi:10.1038/s41598-017-05486-8)
Supplement: Supplementary file 1 — Supplementary info [file 41598_2017_5486_MOESM1_ESM.pdf]

Multimodal imaging quality control of epithelia regenerated with cultured human donor corneal limbal epithelial stem cells

Marco Lombardo,[1,*] Sebastiano Serrao,[1] Vanessa Barbaro,[2] Enzo di Iorio,[3] Giuseppe Lombardo[4,5]

[1] Fondazione G.B. Bietti IRCCS, Via Livenza 3, 00198 Roma, Italy

[2] Fondazione Banca degli Occhi del Veneto, Via Paccagnella 11, 30174 Zelarino (Ve), Italy

[3] Università degli Studi di Padova, Dipartimento di Medicina Molecolare, Via A. Gabelli 63, 35121 Padova, Italy

[4] Consiglio Nazionale delle Ricerche, Istituto per i Processi Chimico-Fisici, CNR-IPCF, Viale F. Stagno D'Alcontres 37, 98158, Messina, Italy

[5] Vision Engineering Italy srl, Via Livenza 3, 00198 Roma, Italy

*Correspondence should be addressed to M.L. (mlombardo@visioeng.it), Fondazione G.B. Bietti IRCCS, Via Livenza 3, 00198 Roma (Italy).

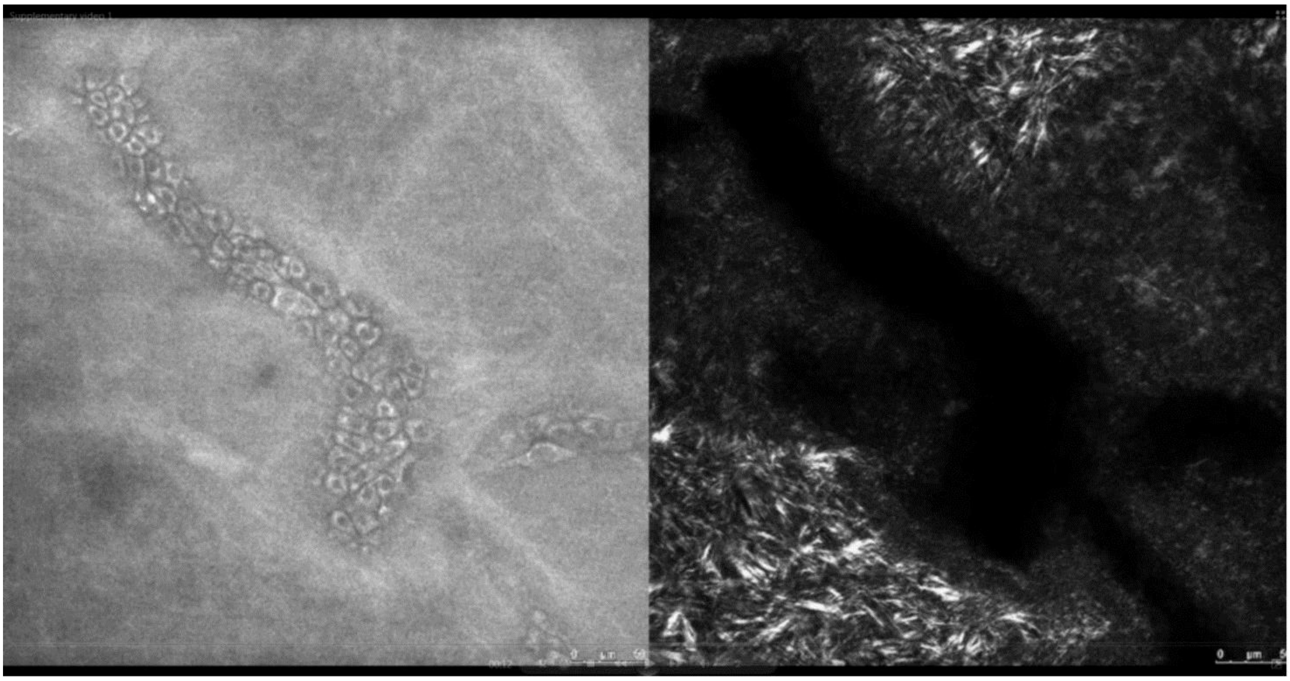

Supplementary video 1

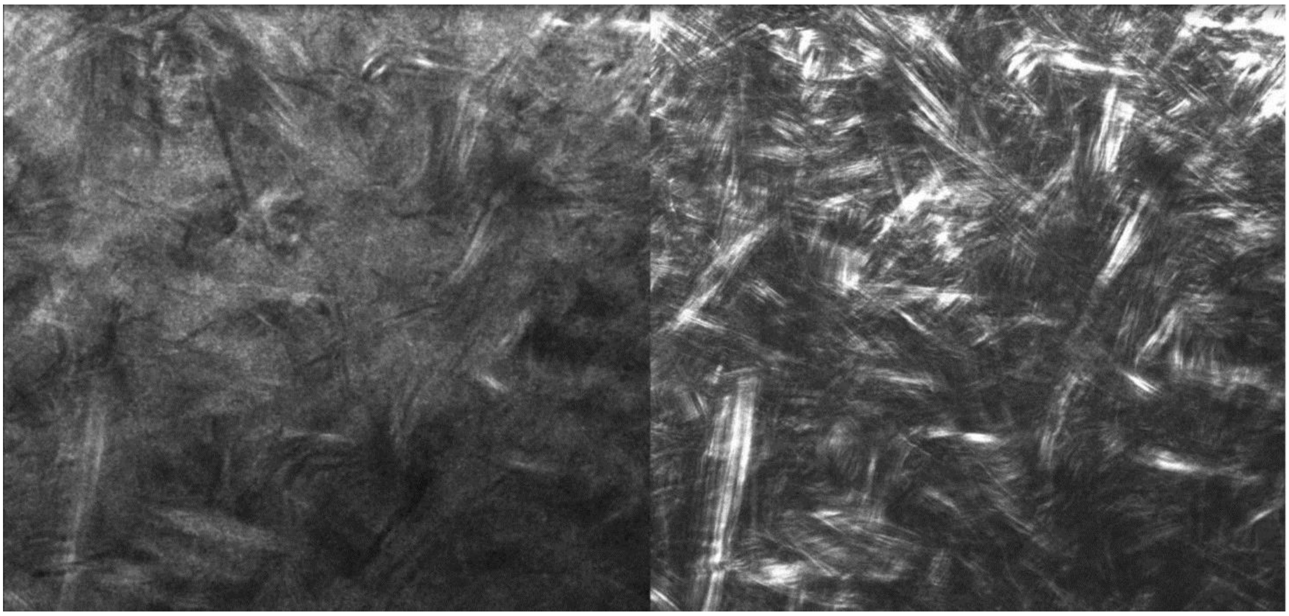

Supplementary video 2

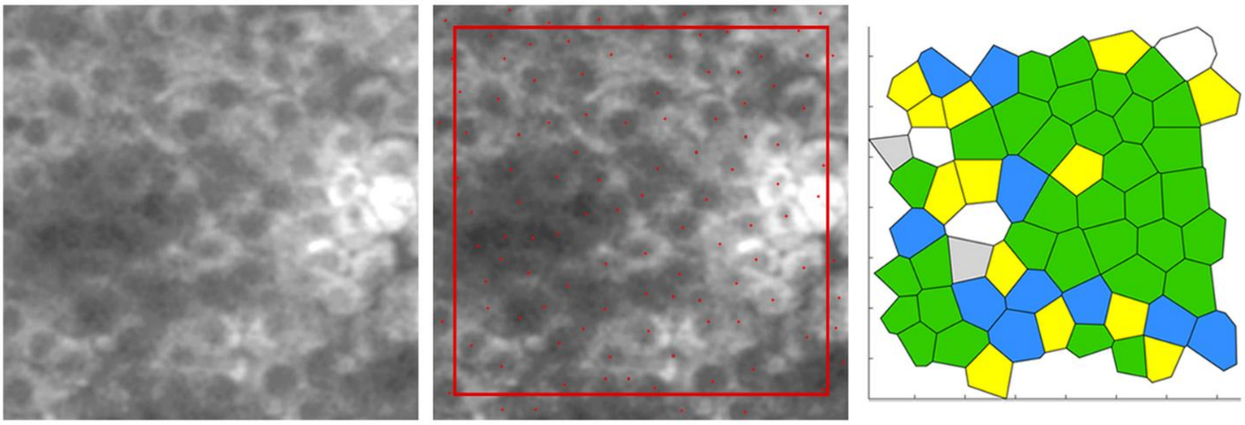

Supplementary figure 1

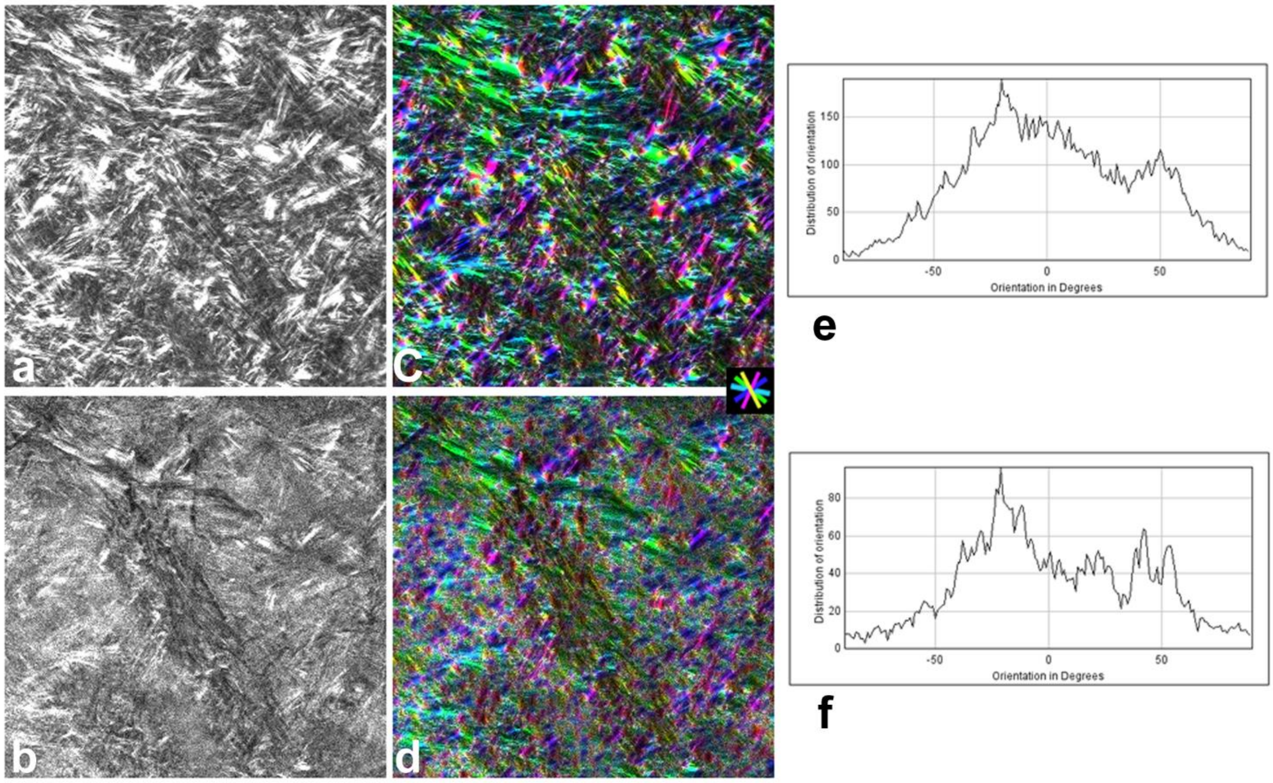

Supplementary figure 2

Legends to supplementary materials

Supplementary video 1 – Video showing the pseudo-palisades of Vogt in the *hemicornea*. Two-photon imaging of the *hemicornea* showing the architecture of the regenerated epithelium and the arrangement of stromal collagen fibers in a representative case. Left panel) two-photon emission fluorescence (TPEF) imaging of the *hemicornea*'s cellular components. The blue arrows highlight the wing cells and the yellow arrows highlight the digital invasion of the basal cuboidal cells into the stroma of the hemicornea. Right panel) forward second harmonic generation (F-SHG) imaging of the *hemicornea*'s extra-cellular components. The regenerated epithelium creates digital invasion in the anterior stroma, resembling the typical architecture of the palisades of Vogt in the human corneoscleral *limbus*. This structure likely represents specialized microenvironment for preserving stem cells in the *hemicornea*. Bars indicate 50 µm.

Supplementary video 2 – Video showing the collagen fiber organization of the most anterior stroma of the *hemicornea*. Two-photon imaging of the *hemicornea* showing the anterior 200 µm anterior stroma in a representative case. Left and right panels) backward (B-SHG) and forward (F-SHG) second harmonic generation imaging of the *hemicornea* respectively; the two modalities produced comparable images of the collagen fibers in the most anterior stroma (<70 µm). Scale bars: 25 µm.

Supplementary figure 1 – Image analysis of cellular components in biomaterial substrates. Main steps in the image analysis method used to investigate the preferred density packing arrangement of corneal epithelial cells (in TPEF images). Left panel) the cell metrics were estimated in 120x120 µm area. Middle panel) the epithelial cells labelled with the automated algorithm; cells whose center was outside the boundary were not labelled. The *nearest neighbour distance* was calculated from point coordinates. Right panel) colour-coded Voronoi tessellation computed from the labelled cells. Each Voronoi cell was colour-coded according to the number of its neighbouring cells: gray = 4n arrangement, yellow = 5n arrangement, green= 6n arrangement;

blue = 7n arrangement and white = 8n arrangement. The Voronoi diagram provides valuable information on the preferred packing arrangement of a cell mosaic, which is associated with the health and integrity of cells.

Supplementary figure 2 – Image analysis of collagen fiber preferred arrangement in the stroma of *hemicornea*. A and B) Forward and backward second harmonic generation (F-SHG and B-SHG) images of stromal collagen fibers in the *hemicornea* at 30 µm depth respectively. C and D) The false colours represent the preferred orientation of collagen fibers lying at the focal plane. E and F) No significant differences in the preferred orientation of collagen fibers were found between forward and backward SHG images.
